# Clinical Factors Associated with Lamina Cribrosa Thickness in Patients with Glaucoma, as Measured with Swept Source Optical Coherence Tomography

**DOI:** 10.1371/journal.pone.0153707

**Published:** 2016-04-21

**Authors:** Kazuko Omodaka, Seri Takahashi, Akiko Matsumoto, Shigeto Maekawa, Tsutomu Kikawa, Noriko Himori, Hidetoshi Takahashi, Kazuichi Maruyama, Hiroshi Kunikata, Masahiro Akiba, Toru Nakazawa

**Affiliations:** 1 Department of Ophthalmology, Tohoku University Graduate School of Medicine, Sendai, Japan; 2 Topcon Corporation, Tokyo, Japan; 3 Department of Retinal Disease Control, Ophthalmology, Tohoku University Graduate School of Medicine, Sendai, Japan; 4 Department of Advanced Ophthalmic Medicine, Tohoku University Graduate School of Medicine, Sendai, Japan; Massachusetts Eye & Ear Infirmary, Harvard Medical School, UNITED STATES

## Abstract

**Purpose:**

To investigate the influence of various risk factors on thinning of the lamina cribrosa (LC), as measured with swept-source optical coherence tomography (SS-OCT; Topcon).

**Methods:**

This retrospective study comprised 150 eyes of 150 patients: 22 normal subjects, 28 preperimetric glaucoma (PPG) patients, and 100 open-angle glaucoma patients. Average LC thickness was determined in a 3 x 3 mm cube scan of the optic disc, over which a 4 x 4 grid of 16 points was superimposed (interpoint distance: 175 μm), centered on the circular Bruch’s membrane opening. The borders of the LC were defined as the visible limits of the LC pores. The correlation of LC thickness with Humphrey field analyzer-measured mean deviation (MD; SITA standard 24–2), circumpapillary retinal nerve fiber layer thickness (cpRNFLT), the vertical cup-to-disc (C/D) ratio, and tissue mean blur rate (MBR) was determined with Spearman's rank correlation coefficient. The relationship of LC thickness with age, axial length, intraocular pressure (IOP), MD, the vertical C/D ratio, central corneal thickness (CCT), and tissue MBR was determined with multiple regression analysis. Average LC thickness and the correlation between LC thickness and MD were compared in patients with the glaucomatous enlargement (GE) optic disc type and those with non-GE disc types, as classified with Nicolela’s method.

**Results:**

We found that average LC thickness in the 16 grid points was significantly associated with overall LC thickness (r = 0.77, *P* < 0.001). The measurement time for this area was 12.4 ± 2.4 minutes. Average LC thickness in this area had a correlation coefficient of 0.57 with cpRNFLT (*P* < 0.001) and 0.46 (*P* < 0.001) with MD. Average LC thickness differed significantly between the groups (normal: 268 ± 23 μm, PPG: 248 ± 13 μm, OAG: 233 ± 20 μm). Multiple regression analysis showed that MD (β = 0.29, *P* = 0.013), vertical C/D ratio (β = -0.25, *P* = 0.020) and tissue MBR (β = 0.20, *P* = 0.034) were independent variables significantly affecting LC thickness, but age, axial length, IOP, and CCT were not. LC thickness was significantly lower in the GE patients (233.9 ± 17.3 μm) than the non-GE patients (243.6 ± 19.5 μm, *P* = 0.040). The correlation coefficient between MD and LC thickness was 0.58 (*P* < 0.001) in the GE patients and 0.39 (*P* = 0.013) in the non-GE patients.

**Conclusion:**

Cupping formation and tissue blood flow were independently correlated to LC thinning. Glaucoma patients with the GE disc type, who predominantly have large cupping, had lower LC thickness even with similar glaucoma severity.

## Introduction

A significant body of evidence suggests that glaucoma should be considered a multifactorial disease. Intraocular pressure (IOP) is the most important treatable risk factor for glaucoma, and accordingly, various strategies have been developed for IOP reduction based on eye drops or minimally invasive tube shunt surgery. However, many non-IOP risk factors have been reported for glaucoma pathogenesis, including aging [1,2], myopia [2,3], family history [4], abnormalities in the lamina cribrosa [5], low ocular perfusion pressure [6,7], oxidative stress [8], inflammation [9], and lifestyle [5,10]. All these risk factors have been found to induce apoptosis of the retinal ganglion cells (RGCs), a key part of glaucoma progression. Previously described molecular mechanisms of RGC death include axonal transport failure, neurotrophic factor deprivation, mitochondrial dysfunction, excitotoxicity, oxidative stress, dysfunctional reactive glia, and the loss of synaptic connectivity [11]. Furthermore, complex combinations of these risk factors can cause glaucomatous visual field defects. As our society ages, it will be increasingly important for ophthalmologists to understand the pathogenesis of glaucoma in order to prevent blindness due to glaucoma from becoming more common. Mechanism-dependent drug discovery will play a key role in achieving this goal.

As glaucoma progresses, the cup of the optic nerve deepens and becomes undermined, and in some cases, the bottom of the cup becomes tilted. The lamina cribrosa (LC), which is located at the bottom of the cup, is composed of a series of sieve-like collagenous plates in the optic nerve head. Postmortem examination of glaucomatous eyes shows that thinning of the LC and deformity of the laminar pores compress the RGC axons, thereby inducing RGC death [12]. Recent technological progress in optical coherence tomography (OCT) has enabled us to visualize the structure of the LC [13,14]. Defects in the LC [5] and thinning of the LC [13,14] have been reported in glaucoma patients. Thus, axonal damage in the LC is considered to play a major role in glaucoma, and LC thickness not only promises to be a biomarker of glaucoma, but also to provide new evidence on the pathogenesis of glaucoma.

Previously reported risk factors for thinning of the LC include high myopia [15] and pseudoexfoliation glaucoma [16]. Eyes with high myopia have a thinner LC than non-myopic eyes, and indeed, LC thickness in highly myopic eyes is similar to non-myopic glaucomatous eyes [15]. Furthermore, eyes with pseudoexfoliation glaucoma have a thinner LC than eyes with primary glaucoma [16]. These findings suggest that LC thickness can be influenced by a variety of risk factors. However, the importance of many other factors, such as cupping, central corneal thickness (CCT), axial length, and blood flow, remains unclear.

In this study, we investigate the association of a wide variety of clinical factors with LC thickness, including age, IOP, CCT, axial length, various measurement parameters of disc cupping, and blood flow. In order to recruit a sufficient number of glaucoma patients, we developed a new, simplified method to measure LC thickness. Previously, we developed custom software to quantitatively measure LC thickness [14]. However, using this software was time-consuming, limiting its usefulness and restricting the number of glaucoma patients that could be studied. The new version of the software used in the present study was simplified and quicker to use, allowing us to save time during examinations and investigate in greater detail the factors influencing LC thickness in glaucoma.

## Materials and Methods

### Subjects

This retrospective study comprised a total of 150 eyes from 150 cases, including 22 normal subjects, 28 preperimetric glaucoma (PPG) patients, and 100 open-angle glaucoma patients (mean age 60.8 ± 9.7, male: female = 78: 72). The inclusion criteria were good visual acuity (BCVA; best-corrected decimal visual acuity > 0.4) and a normal axial length (less than 28 mm). Patients with ocular diseases other than OAG, with severe myopia (< -6 diopter), without visible lamina pores, and with tilted myopic discs, or with systemic diseases affecting the visual field were excluded from this study.

Baseline clinical parameters were recorded from each patient, including age, refractive error, BCVA, IOP, CCT, Humphrey Field Analyzer (HFA; Carl Zeiss Meditec, Dublin, California, USA)-measured mean deviation (MD), axial length, and blood pressure. BCVA was measured with a standard Japanese decimal visual acuity chart, which was converted to the logarithm of the minimum angle of resolution (logMAR). IOP was examined with Goldmann applanation tonometry on the same day as the OCT examination, without interrupting the use of OAG medication. Before pupil dilation, slit-lamp biomicroscopy and gonioscopy were performed to diagnose a primary open angle. Anterior-segment OCT (CASIA, Tomey Corporation, Nagoya, Japan) was used for measuring CCT. Following pupil dilation with tropicamide (Midrin M, Santen Pharmaceutical, Osaka, Japan), stereoscopic examination of the optic nerve head was performed, fundus and disc photographs were taken and ocular biometry (IOL Master; Carl Zeiss Meditec) was performed with full pupil dilation. All the examination data were obtained within a 2-month period.

Mean deviation (MD) values were obtained with the HFA using the Swedish interactive threshold algorithm (SITA)-standard strategy of the 24–2 program. Only reliably measured data were used (i.e., with a fixation loss < 20%, false-positive errors < 15%, and false-negative errors < 33%). A glaucomatous visual field was defined, according to the Anderson-Patella criteria, [17] by one or more of the following: (1) a cluster of three points with probabilities of < 5% on the pattern deviation map in at least one hemifield (including ≥ 1 point with probability of < 1% or a cluster of two points with a probability of < 1%, (2) glaucomatous hemifield test results outside the normal limits or (3) a pattern standard deviation beyond 95% of normal limits, as confirmed in at least 2 reliable examinations. PPG was defined by evidence of structural changes such as cupping, rim notching, and retinal nerve fiber layer defects with no evidence of any glaucomatous visual defects, as described above.

Both systemic blood pressure and pulse rate were measured in the left brachial artery at the height of the heart with an automated blood pressure (BP) monitor (HEM-759E; Omron Corporation, Kyoto, Japan) with the subject in a sitting position. Ocular perfusion pressure (OPP) was defined as the mean arterial pressure (MAP, at eye level) minus IOP. MAP = diastolic BP +1/3(systolic BP–diastolic BP).

We assessed ocular blood flow by measuring mean blur rate (MBR) in the optic nerve head using laser speckle flowgraphy (LSFG-NAVI, Softcare Co., Ltd, Japan), which uses the laser speckle phenomenon to detect and quantify ocular circulation in a noninvasive manner, both overall and separately in the vessel and tissue areas. Tissue-area MBR in the optic nerve head has been shown to be linearly correlated with capillary blood flow and was used for the analysis in this study.

This study adhered to the tenets of the Declaration of Helsinki, and the protocols were approved by the Clinical Research Ethics Committee of the Tohoku University Graduate School of Medicine (study 2014-1-805). Participants provided their written informed consent to participate in this study. The ethics committees approved this consent procedure.

### Development of new, simplified software to measure LC thickness

**Fig 1:**
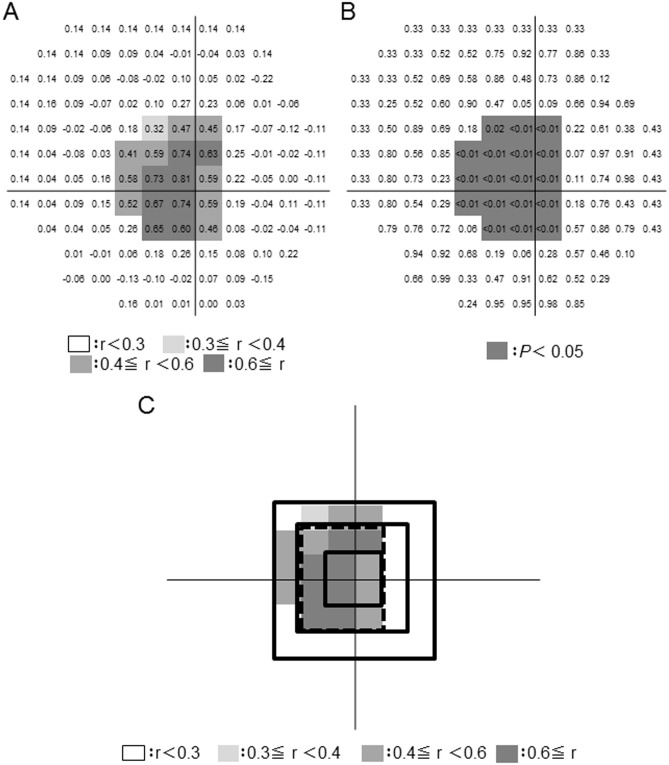
Distribution of the correlation between local and overall lamina cribrosa (LC) thickness in a grid superimposed on the LC. (A) The numbers in the grid cells represent Spearman’s rank correlation coefficient. The cells highlighted in gray had a higher correlation coefficient. (B) The numbers in the grid cells represent the *P* value. The cells highlighted in gray were significantly correlated (*P* value < 0.05). (C) Map showing the position of 4 areas of high correlation, comprising 4, 12, 16, and 36 grid cells, respectively.

One of the goals of this study was to identify sub-regions of the LC that could accurately represent the thickness of the entire LC. As a first step, we used previously described custom software to measure and segment the structure of the LC in 3D, in a process based on swept-source OCT (SS-OCT; Topcon) images.[14] Briefly, we first obtained a 3 x 3 mm cube scan of an area centered on the optic disc and derived 12 reconstructed radial B-scans from the data. We then manually marked the edges of the Bruch’s membrane opening (BMO) in order to identify the BMO center. Next, the software synchronized the reconstructed B-scan images and a set of en-face images of the LC. The anterior and posterior borders of the LC were defined in the reconstructed B-scan images as the points where the LC pores became visible. We marked a series of points on these borders, and used the points to calculate splines and automatically segment the LC in 3D. An important element of our technique was a method to overcome the influence of the shadows of the large vessels, which are known to interfere with efforts to precisely examine the LC surface. In this method, we manually identified the reliably-measured area in each B-scan image by selecting the area with the highest signal intensity, and restricted the calculation of average LC thickness in the 3D-segmented LC plate to the reliably-measured area [14]. After preparation of the data was complete, we attempted to identify sub-regions of the LC where local thickness was correlated with overall thickness. To do this, 16x16 grid was superimposed into the LC area which covered area of 2.8 x 2.8-mm. Average LC thickness was measured for each grid. We then determined the correlation between LC thickness in each grid cell and overall LC thickness (Fig 1).

**Fig 2:**
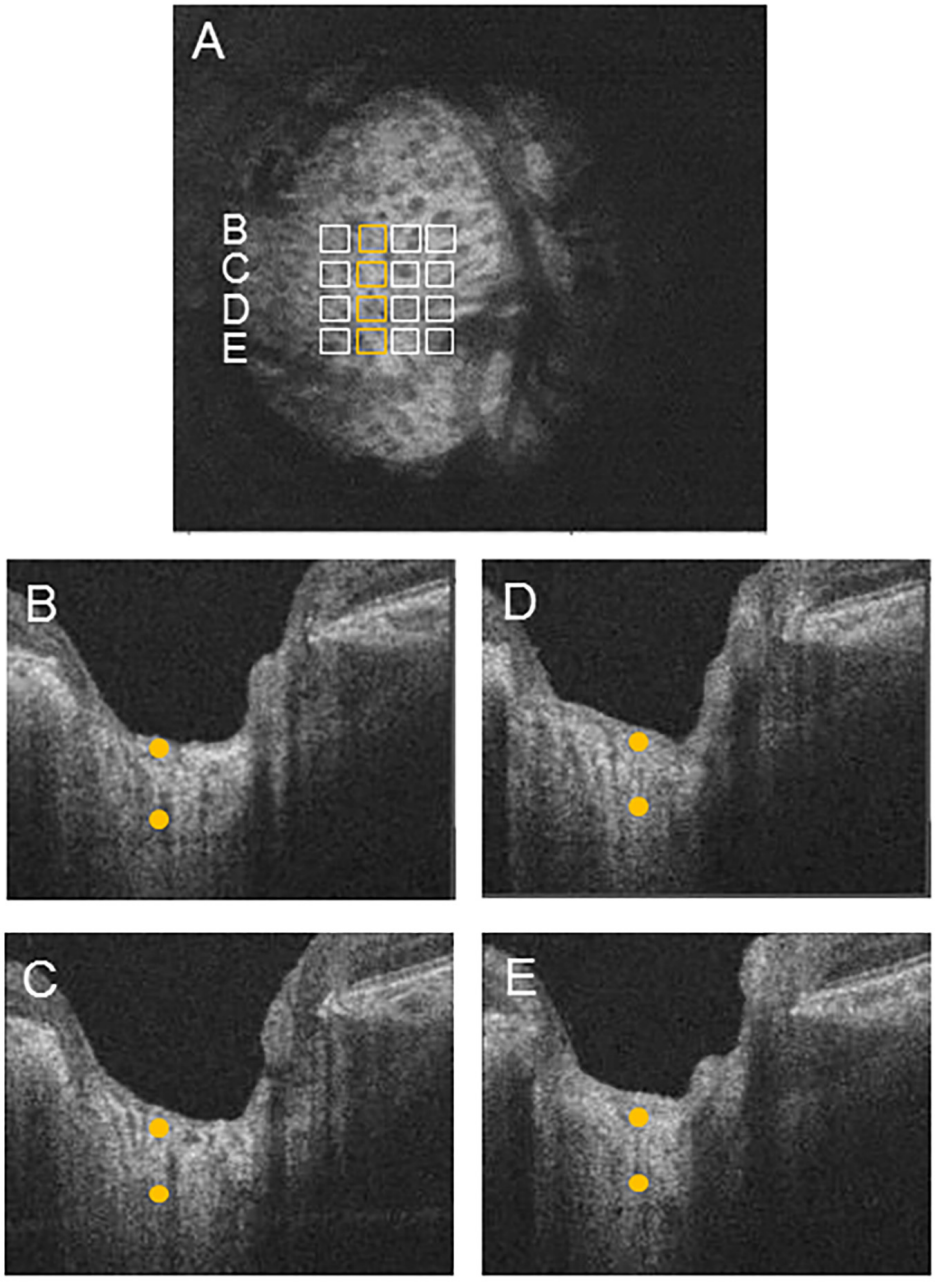
Simplified software technique for measuring lamina cribrosa (LC) thickness in a 4 x 4 grid. (A) 4 x 4 grid superimposed on an en-face image of the LC, showing the position of the B-scan images below. (B-E) Horizontal cross-sectional B-scan images, with orange dots indicating the anterior and posterior borders of the LC. Average LC thickness was defined as the average thickness in the 16 grid cells.

Next, we calculated the correlation between overall LC thickness and the average of local, point-by-point LC thickness in each of 4 predefined areas, which comprised 4, 12, 16, or 36 grid cells (Fig 1C). We found that the highest correlation was in the area comprising 16 grid cells. We thus decided to use this region to represent the overall LC, and chose it for inclusion in a new, simplified version of our custom software for measuring average LC thickness. In this version of the software, BMO edge was set manually by clicking the 12 reconstructed B-scan images and the center of the BMO was determined by the centroid of the BMO edges. A 4 x 4 grid was superimposed over the image, corresponding to the 16-grid-cell area identified as highly correlated with the overall LC. The grid, in which each square represented a 175 μm square region of the LC, was centered on the centroid of BMO with Custum-made OCT En-Face Viewer for LC thickness software (Fig 2).

**Fig 3:**
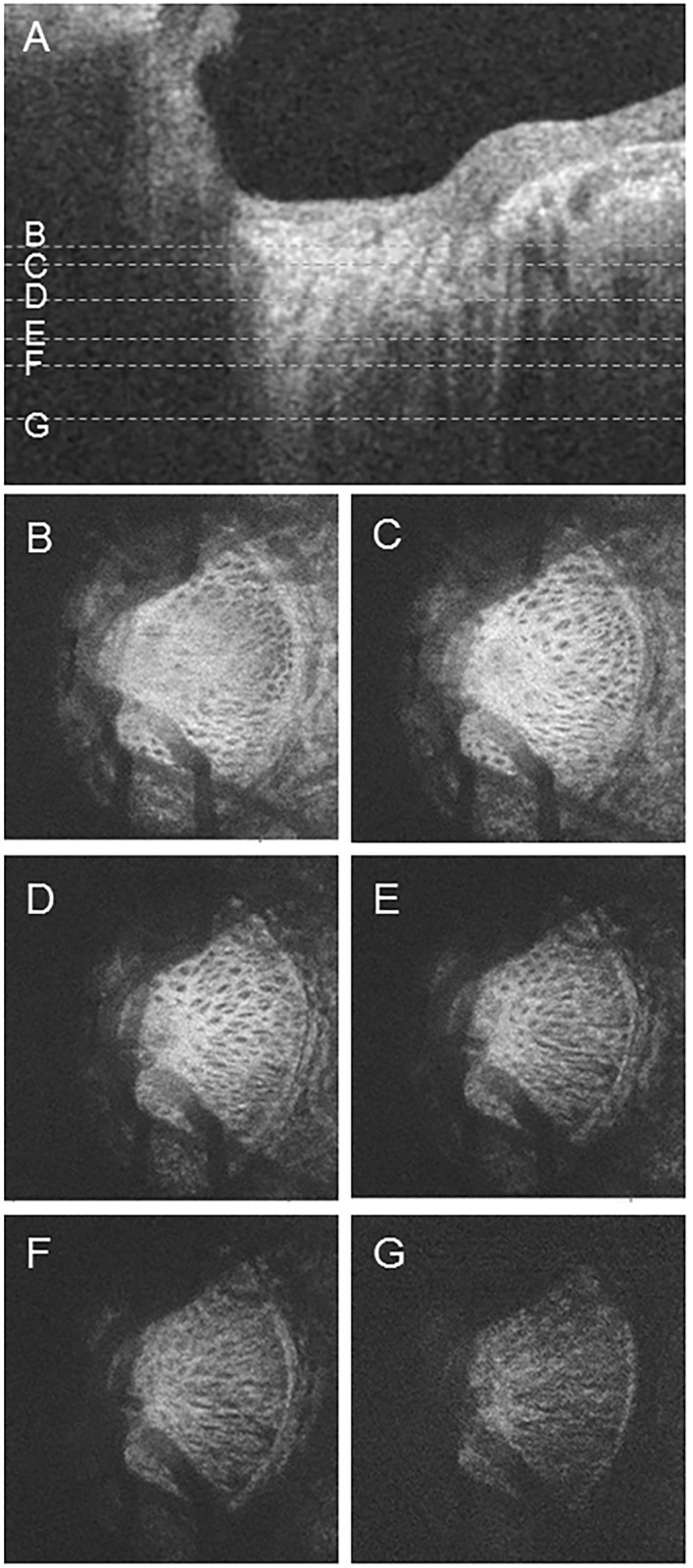
B-scan and en-face images. (A) B-scan image. The dotted lines (B-G) indicate the position of the en-face images below. (B) Upper area of the lamina cribrosa (LC). (C) Upper border of the LC. (D, E) Centerline of the LC. (F) Lower border of the LC. (G) Lower area of the LC.

Next, we measured average LC thickness in each cell of the 16-grid-cell area after marking the anterior and posterior borders of the visible LC pores in the B-scan images (Fig 3). Finally, we determined the cell-by-cell correlation with HFA MD, circumpapillary retinal nerve fiber layer thickness (cpRNFLT), the vertical cup/disc (C/D) ratio, and tissue MBR. If a grid cell was over a vessel, making it hard to visualize the LC pores, we excluded that grid cell from the calculations.

### Classification of disc appearance

**Fig 4:**
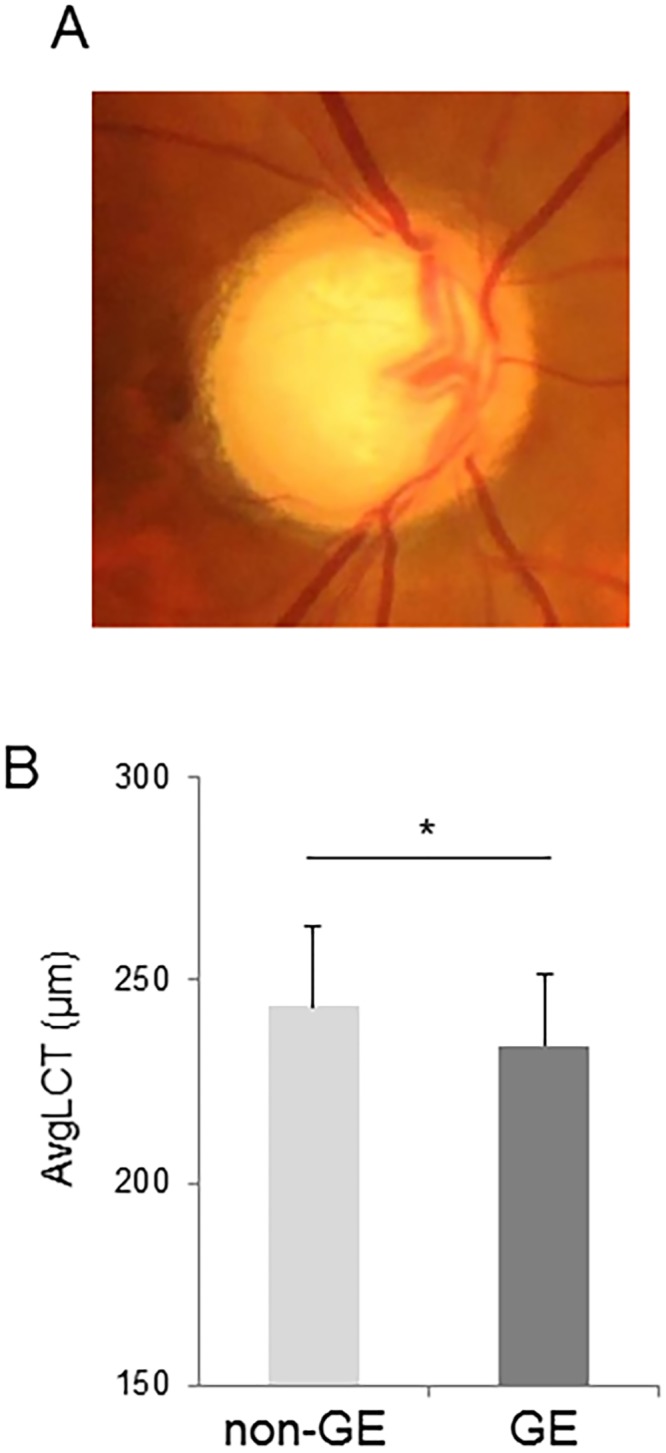
Difference in the association between average lamina cribrosa thickness (avgLCT) and HFA MD in glaucoma patients with generalized enlargement (GE)-type discs and non-GE discs. (A) Representative appearance of a GE disc. (B) Comparison of avgLCT in patients with non-GE and GE discs.

In order to better understand the relationship between cupping formation and LC thickness, we classified the patients into two groups based on optic nerve head morphology: one group contained patients with generalized enlargement (GE)-type discs, and the other group contained patients with all other disc types (the non-GE group). This classification was performed based on the method reported by Nicolela and Drance. [18]. GE discs have large, deep cupping, especially in the nasal area, making them the most suitable type for investigating the role of cupping and thinning of the LC (Fig 4A). Three glaucoma specialists performed the disc type classification of the 150 eyes independently. In 35 cases (23%), eyes were excluded because the examiners did not agree on a classification or considered a particular optic disc too hard to classify. Finally, the non-GE group (43 cases) and the GE group (44 cases) were analyzed statistically.

### Analysis

All statistical analyses were performed with JMP software (version 10.0.2, SAS Institute Japan Inc., Tokyo, Japan). We investigated the association between LC thickness in 4 different regions of the LC, comprising 4, 12, 16, or 36 cells of a superimposed grid, and overall LC thickness in order to select the most appropriate region for simplified measurement software. We then used this software to calculate average LC thickness and used Spearman's rank correlation coefficient to compare it with HFA 24–2 measurements of MD, cpRNFLT, the vertical C/D ratio, and tissue MBR. We performed a multiple regression analysis to investigate the relationship between LC thickness and age, axial length, IOP, MD, the vertical C/D ratio, CCT, and tissue MBR. Differences were considered significant at *P* < 0.05.

Comparisons of more than three groups were made with the Kruskal-Wallis test, followed by the Steel-Dwass test. Comparisons of pairs of groups were made with the Mann-Whitney U test. The Chi-square test was used for frequency data on the sex ratio. The Z test was performed to determine the significance of differences in correlation coefficients between the GE and non-GE disc groups.

## Results

### Development of simplified software

**Fig 5:**
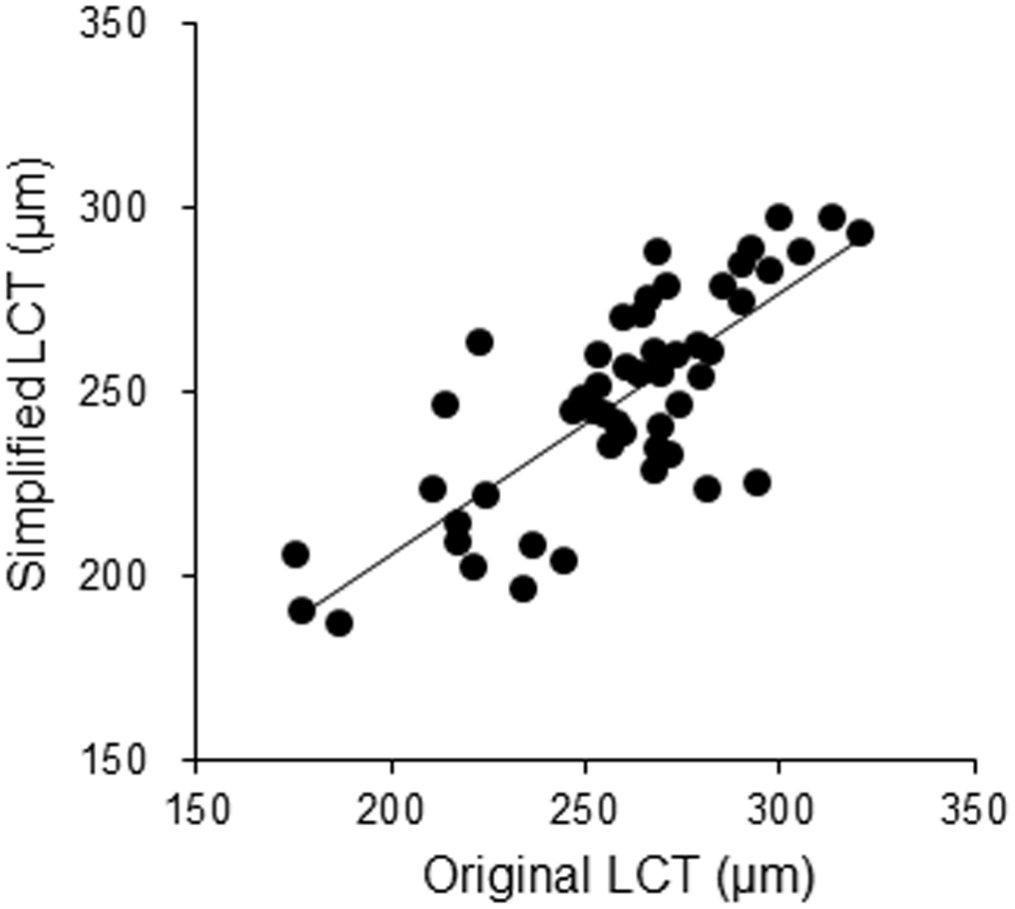
The correlation between measurements of lamina cribrosa thickness (LCT) made with the original and simplified versions of the software. Scatter plot graph showing the correlation of LCT measurements made with the original and simplified versions of the software. Note: the correlation coefficient between LCT measurements made with the original and simplified versions was 0.71 (*P* < 0.001).

In order to investigate factors influencing thinning of the LC, we developed simplified software for measuring LC thickness. To reduce the examination time, we tried to limit the amount of user interaction demanded by the software. To develop this software, we first determined which sub-region of the LC was most significantly correlated with overall LC thickness in the reliably-measured area. We superimposed a 16 x 16 grid on the overall LC, and calculated the correlation coefficient between LC thickness in each cell of the grid and overall LC thickness. Fig 1 shows the correlation coefficient (Fig 1A) and *P* value (Fig 1B) for each grid cell. Noting that the central LC had a greater number of highly correlated grid cells, we defined 4 overlapping local regions of the LC, containing 4, 12, 16 or 36 grid cells. We investigated the correlation coefficient between the average LC thickness within these regions and overall LC thickness (Fig 1C). The 4-grid-cell area had a correlation coefficient of 0.70 and average LC thickness of 254.3 ± 44.1 μm, the 12-grid-cell area had a correlation coefficient of 0.74 and average LC thickness of 252.8 ± 39.0 μm, the 16-grid-cell area had a correlation coefficient of 0.77 and average LC thickness of 261.0 ± 42.3 μm, and the 36-grid-cell area had a correlation coefficient of 0.69 and average LC thickness of 212.5 ± 35.4 μm (all *P* < 0.001). Thus, the 16-grid-cell area, which covered a 700-μm square region at the center of the LC, was the most significantly correlated with the overall LC. The average time to measure LC thickness in this area with the new software was 12.4 ± 2.4 minutes, compared to the 40.3 ± 4.3 minutes required by the older version of the software. Thus, we were able to measure average LC thickness in significantly less time with the new, simplified software, even though it was not capable of measuring the volume of the LC. The reproducibility of LC thickness measurements made with the new software was good, with an intraclass correlation coefficient (ICC) of 0.86. The correlation coefficient between measurements of LCT made with the original and simplified versions of the software was 0.71 (*P* < 0.001) (Fig 5). Thus, we continued our analysis using the new version of the software.

### Correlation between glaucoma stage and LC thickness

Table 1 summarizes the clinical characteristics of the subjects in this study. There were no significant differences in age, sex, axial length, systemic blood pressure, ocular perfusion pressure, or pulse rate (all *P* > 0.05).

**Table 1:** Demographic data for this study. D: diopter. IOP: intraocular pressure.

	Normal (n = 22)	PPG (n = 28)	OAG (n = 100)	P Value
Age (years)	58.7 ± 9.7	62.2 ± 7.2	60.9 ± 10.2	NS*
Male: female	10: 12	9: 19	54: 46	NS†
Axial length (mm)	24.2 ± 1.2	24.2 ± 1.1	24.6 ± 1.2	NS*
IOP (mm Hg)	14.7 ± 2.8	14.8 ± 1.7	17.0 ± 4.4	0.012*
CCT (μm)	539.9 ± 30.4	518.5 ± 35.6	507.6 ± 36.4	0.018*
MD (dB)	0.98 ± 0.60	0.70 ± 0.91	-7.17 ± 7.18	<0.001*
cpRNFLT (μm)	113.2 ± 11.0	101.7 ± 8.5	86.2 ± 14.8	<0.001*
Vertical C/D ratio	0.64 ± 0.10	0.71 ± 0.09	0.89 ± 0.09	<0.001*
Tissue MBR (au)	10.9 ± 1.8	9.8 ± 1.7	9.7 ± 2.4	0.022*
Systolic BP(mmHg)	130.1 ± 13.7	128.8 ± 19.0	121.1 ± 14.0	NS*
Diastolic BP (mmHg)	72.1 ± 9.2	76.2 ± 9.6	72.6 ± 10.1	NS*
Mean arterial BP (mmHg)	91.4 ± 9.6	93.8 ± 11.9	88.7 ± 10.1	NS*
OPP (mmHg)	79.9 ± 13.0	79.9 ± 12.5	75.6 ± 9.5	NS*
Pulse rate (beats/min)	76.8 ± 13.5	75.9 ± 5.2	71.5 ± 12.9	NS*

CCT: central corneal thickness. MD: Humphrey field analyzer-measured mean deviation. cpRNFLT: circumpapillary nerve fiber layer thickness. MBR: mean blur rate. BP: blood pressure. OPP: Ocular perfusion pressure. PPG: preperimetric glaucoma. OAG: open-angle glaucoma. The Kruskal-Wallis test was used to determine *P* values for age, refractive error, IOP, CCT, MD, cpRNFLT, vertical C/D ratio, BP and pulse rate, and the Chi-square test was used to determine *P* values for sex.

* Differences between groups were assessed with the Kruskal-Wallis test.

^†^ The Chi-square test was used to analyze frequency data on sex.

**Fig 6:**
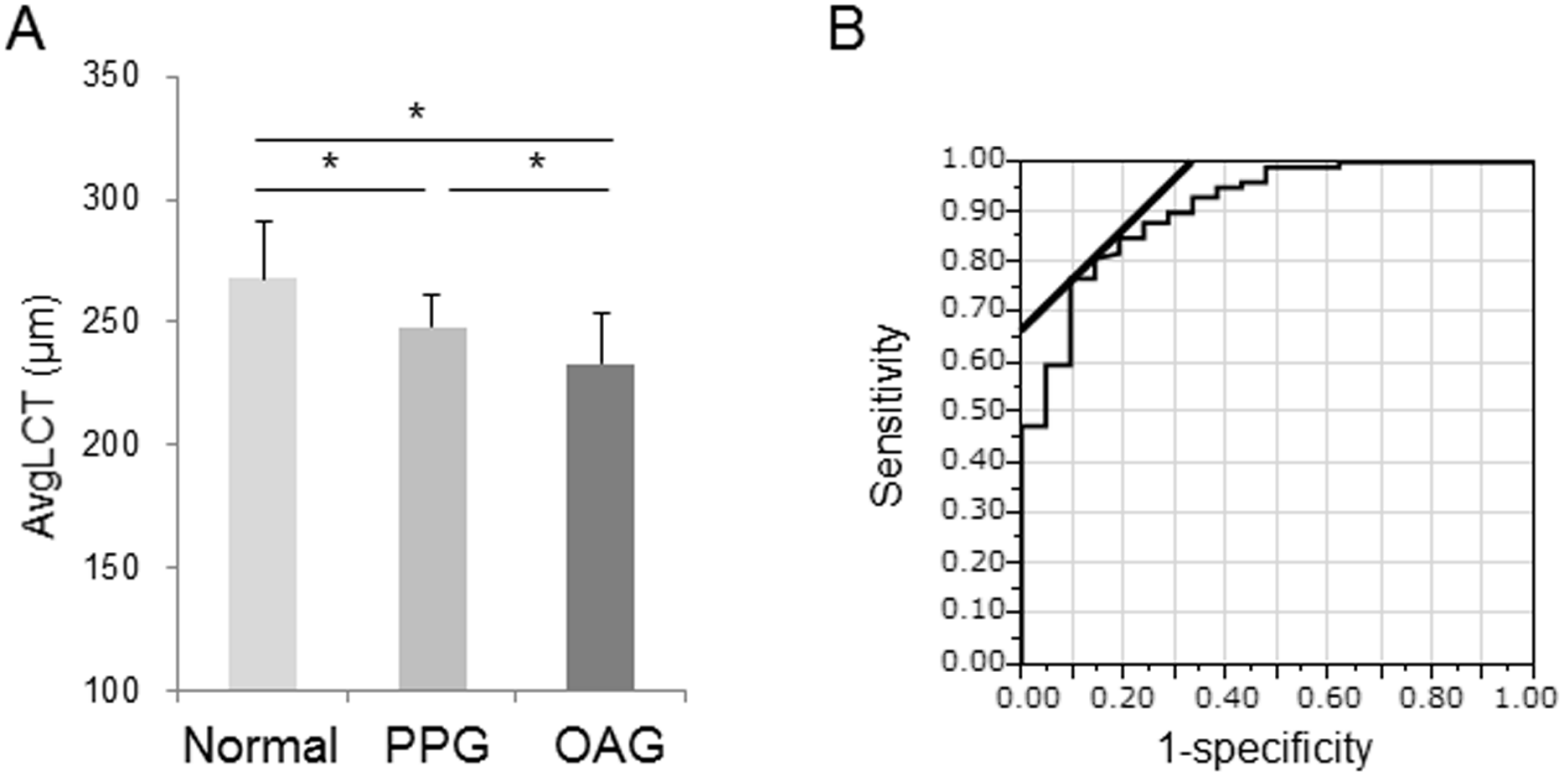
AvgLCT measurements made with the simplified version of the software. (A) Bar graph indicating LC thickness in patients with different stages of glaucoma (normal: n = 22, PPG: n = 28, OAG: n = 100). Note: there were significant differences between these groups (Kruskal-Wallis test followed by Steel-Dwass test). *: *P* < 0.01. (B) ROC curve. The area under the ROC curve was 0.91, with a cutoff value of 246.3 μm.

The average thickness of the LC differed significantly in the groups (normal: 268 ± 23 μm, PPG: 248 ± 13 μm, and OAG: 233 ± 20 μm), and decreased significantly with the stage of glaucoma (normal vs. PPG, PPG vs. OAG, and normal vs. OAG; all *P* < 0.01) (Fig 6A). The area under the ROC for differentiating normal subjects and glaucoma patients was 0.91 (Fig 6B) and the cutoff value for LC thickness was 246.3 μm (sensitivity: 0.77; specificity: 0.90).

**Fig 7:**
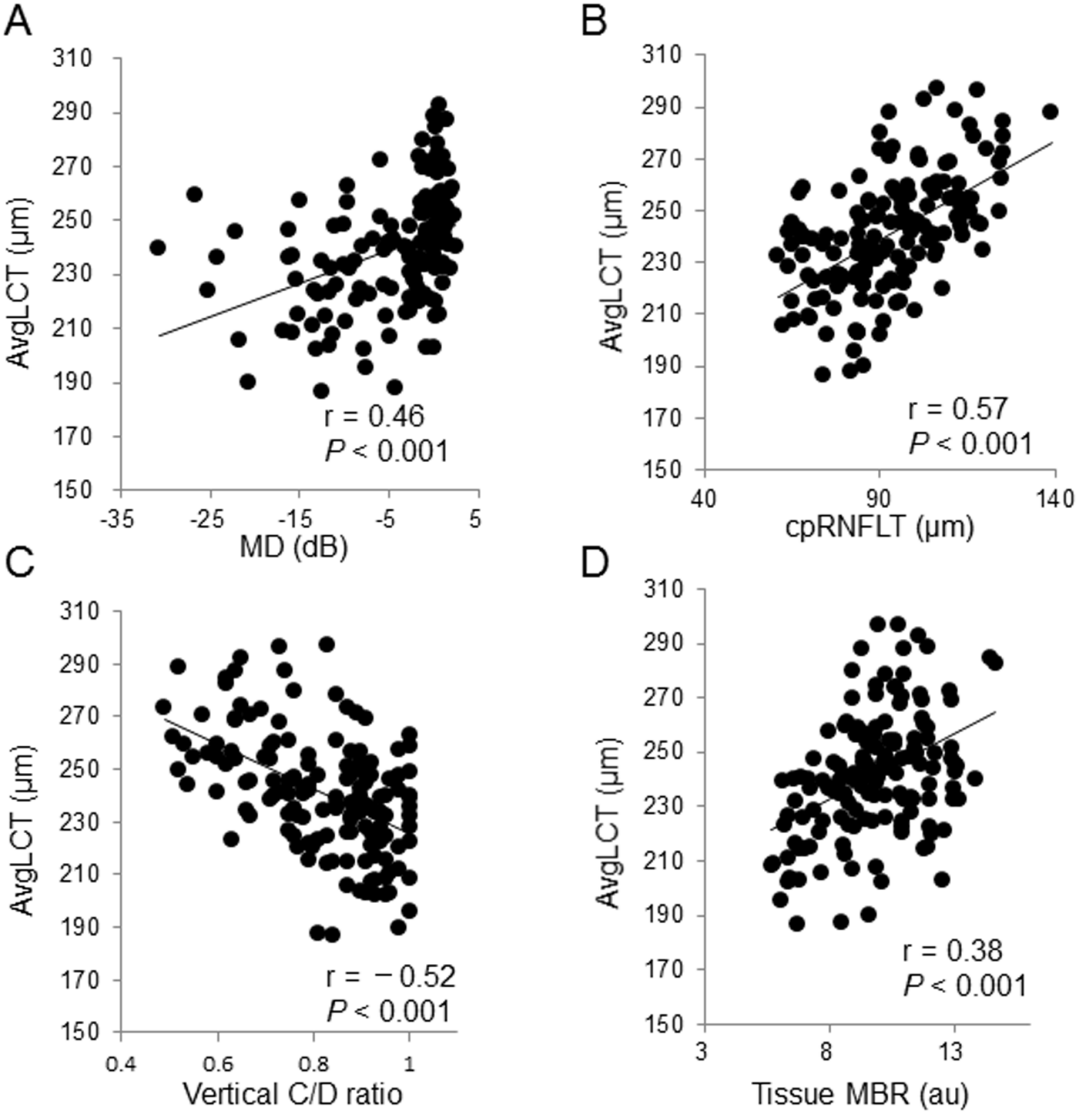
Association between glaucoma parameters and average thickness of the LC (AvgLCT). (A) MD (B) cpRNFLT (C) vertical C/D ratio (D) tissue MBR.

The correlation coefficient with LC thickness was 0.46 for MD (n = 150, *P* < 0.001), 0.57 for cpRNFLT (*P* < 0.001), -0.52 for the vertical C/D ratio, and 0.38 for tissue MBR (*P* < 0.001) (Fig 7). These data suggest that LC thickness measurements made with the simplified software are correlated to the vertical C/D ratio and tissue blood flow, and that they may be a valuable way to assess glaucoma severity.

### Multiple regression analysis of possible influencing factors

In order to investigate possible influencing factors on LC thinning, we performed a multiple regression analysis with the following parameters: age, axial length, IOP, MD, the vertical C/D ratio, CCT, and MBR. We found that MD, the vertical C/D ratio, and tissue MBR were independent variables significantly affecting LC thickness (β = 0.29, *P* = 0.013; β = -0.25, *P* = 0.020; and β = 0.20, *P* = 0.034, respectively), but that age, axial length, IOP, and CCT were not (Table 2). These data suggest that cupping formation and blood flow are factors influencing thinning of the LC.

**Table 2:** Multiple regression analysis of independent variables affecting lamina cribrosa thickness.

Dependent Variable	β	P Value
Age	-0.01	0.897
Axial length	0.08	0.359
IOP	-0.07	0.446
MD	0.29	0.013
Vertical C/D ratio	-0.25	0.020
CCT	-0.03	0.719

β is the standardized coefficient. Differences were considered significant at *P* < 0.05.

### LC thickness in patients with the GE disc type

Cupping formation was strongly correlated to LC thickness, which prompted us to investigate LC thickness in patients with the GE disc type. Patients with this disc type have a significantly larger vertical C/D ratio than those with other types, even when there is a similar level of damage from glaucoma [19–21]. Classifying patients by disc type revealed a significant difference in the vertical C/D ratio (*P* = 0.021), but not in age, the sex ratio, axial length, IOP, MD, CCT, or cpRNFLT, between patients with the GE disc type and those with other types (Table 3).

**Table 3:** Clinical characteristics of patients with non-GE and GE discs.

	Non-GE (n = 43)	GE (n = 44)	P Value
Age (years)	62.0 ± 11.0	60.0 ± 8.9	NS*
Male: female	19: 24	23: 21	NS†
Axial length (mm)	24.4 ± 1.1	24.1 ± 1.0	NS*
IOP (mm Hg)	15.2 ± 4.1	16.4 ± 3.7	NS*
CCT (μm)	509.1 ± 28.0	513.2 ± 37.5	NS*
MD (dB)	-3.54 ± 4.46	-3.45 ± 5.47	NS*
cpRNFLT (μm)	94.3 ± 16.0	90.9 ± 14.3	NS*
Vertical C/D ratio	0.79 ± 0.10	0.85 ± 0.11	0.021*

Differences were considered significant at *P* < 0.05. NS: not significant.

* Differences between groups were assessed with the Kruskal-Wallis test.

^†^ The Chi-square test was used to analyze frequency data on sex.

We found that LC thickness in the GE disc-type patients (233.9 ± 17.3 μm) was significantly lower than in the non-GE disc-type patients (243.6 ± 19.5 μm, *P* = 0.040) (Fig 4B). The correlation coefficient between MD and LC thickness was 0.58 (*P* < 0.001) in the GE disc-type patients and 0.39 (*P* = 0.013) in the non-GE disc-type patients. The Z-score for the correlation coefficient was 2.28, showing that the correlations in the two groups were significantly different. These data suggest that the correlation between LC thickness and MD was significantly stronger in glaucoma patients with larger cupping of the disc.

## Discussion

To investigate risk factors associated with thinning of the LC, we began by developing new software to evaluate LC thickness quickly and accurately, as this would allow us to include a larger number of cases. We based this new software on an analysis of the LC that revealed LC sub-regions where local thickness was strongly correlated to overall thickness. The region with the strongest correlation was a central area comprising 16 test points, and we thus chose this area for inclusion in our new software. The average time to measure LC thickness in this area was 12.4 ± 2.4 minutes. We examined 150 patients and found that LC thickness was significantly lower in the PPG and OAG stages of glaucoma. Furthermore, the association between MD and LC thickness was significant, with a correlation coefficient of 0.46. A multiple regression analysis also showed that average LC thickness was associated with the C/D ratio, blood flow, and visual field, but not with age, CCT, or axial length. These data suggest that LC thinning progresses with visual field loss, and that cupping formation and tissue blood flow are risk factors. Additionally, we found that patients with the GE optic disc type, which has large cupping, had lower LC thickness than other patients, even when HFA-measured MD was similar. Thus, we found that LC thickness was an excellent structural biomarker for glaucoma diagnosis, and that it promises to help future investigations into the pathophysiology of glaucoma.

We found that the thickness of a central region of the LC was closely correlated to overall LC thickness, and took advantage of this to save time during examinations by choosing 16 test points centered on the circular BMO. The LC plays a key role in glaucoma pathogenesis, because its superior and inferior sections have large pores that are vulnerable to deformation. Nevertheless, our study shows that measuring the central region of the LC is sufficient to estimate its overall thickness. This was a valuable finding, because current methods of OCT examination are unable to obtain clear images of areas under vessels or in the undermined cupped area because of an insufficient signal. Even new techniques capable of attenuating and removing shadows and enhancing contrast, although significant improvements, are still unable to obtain blur-free data from underneath vessels, and these data are thus unsuitable for the accurate evaluation of the LC pores [22]. Therefore, measuring the central area of the LC is currently a more reliable and accurate way of assessing the LC.

Previous reports have described several different methods for measuring LC thickness that were based on SD-OCT [23], alone or in combination with enhanced depth imaging (EDI) [24]. Most of these methods used 3 horizontal B-line scans through the optic nerve head [13,25]. The main advantage of this method is that it can measure the prelaminar region of the optic nerve and LC thickness simultaneously, and its main disadvantage is that it cannot adequately identify the posterior border of the LC. Therefore, many recent studies that used this method have focused on structural evaluations of the prelaminar area [21,26]. By contrast, the current study was able to clearly reveal the laminar pores of the LC in C-scan images. The resulting LC thickness data were reproducible and can be considered valid due to their high correlation to glaucoma damage. Thus, this study revealed factors influencing LC thickness, and we believe that the methods described here should enhance the evaluation of glaucomatous structural changes in the LC.

A multiple regression analysis showed that MD, the vertical C/D ratio, and tissue MBR were independently correlated to LC thickness, but that age, CCT, and axial length were not. A previous study used SD-OCT to demonstrate that central LC thickness increased significantly with age in healthy human eyes, but that CCT and axial length did not [25]. One explanation for this discrepancy with our findings on LC thickness and age is that even though LC thickness may increase with age in healthy eyes, in eyes with glaucoma, the LC becomes thinner. Furthermore, CCT has been found to correlate to anterior scleral thickness in NTG patients, but not in patients with ocular hypertension or POAG [27]. In healthy subjects, meanwhile, CCT was found to be not significantly correlated with LC thickness, peripapillary scleral thickness, or the shortest distance between the intraocular space and the cerebrospinal fluid space [28]. Similarly, we found that there was no correlation between CCT and LC thickness. Taken together, these results suggest that there is no anatomic correspondence between CCT and LC thickness in patients with NTG.

The independent association of the vertical C/D ratio and LC thickness was a reasonable finding, since a large vertical C/D ratio is included in the definition of glaucomatous optic neuropathy. Recent work has shown that low LC thickness and a high degree of LC displacement have a significant influence on the progression of RNFL thinning [29], and that central LC depth is associated with a higher translaminar pressure difference and gradient in healthy human eyes [30]. Thus, cupping formation and subsequent thinning of the LC may induce axonal damage in the retinal nerve fibers. However, cupping depth varies significantly according to the shape of the disc. In particular, GE-type discs have a larger, deeper cup than other types of disc [19–21], which prompted us to divide patients into groups based on the presence or absence of a GE disc, and compare LC thickness between them. This comparison showed that LC thickness was significantly lower in the GE group than the non-GE group, and that the correlation between LC thickness and HFA MD was stronger in the GE group than the non-GE group. These data strengthen our finding that cupping influences thinning of the LC in patients with OAG.

Our multiple regression analysis also showed that LC thickness and tissue MBR were independently correlated. MBR, a parameter measuring ocular blood flow, has been shown to be valid for inter-individual comparisons [31–33]. Furthermore, disc MBR has previously been found to be strongly correlated to blood flow around the area of the LC in studies based on experimental glaucoma, [31] making the association that we observed between MBR and LC thickness very interesting. Recently, we showed that systemic oxidative stress was correlated to MBR in early glaucoma [34]. In patients with PPG, we found that vessel-area MBR had already decreased in PPG patients [35], while OCT angiography has shown that vessel index is associated with PSD and early parameters of the visual field [36]. Taken together, these results show that cupping formation, the disappearance of the capillaries in the optic nerve head, and thinning of the LC are detectible in the earliest stages of glaucomatous visual field loss.

This study had several limitations. We were not able to find an association between reduced LC thickness and myopia, even though earlier reports have linked these conditions [15]. In the current study, patients with severe myopia were excluded. Additionally, due to the nature of our new examination technique, patients without visible pores in the LC were excluded. As a result, the average refractive error of the patients included in this study was -0.83 diopters. Furthermore, patients with tilted myopic discs tended to be excluded, because the technical characteristics of OCT prevent the clear imaging of tilted optic discs. Nevertheless, myopia is a major risk factor for glaucoma,[2] and the clinical differentiation of myopic patients with glaucoma from those without glaucoma is important. Further technical development is thus needed to enable the clear imaging of tilted discs.

## Conclusion

The main finding of this study was that LC thickness was correlated not only with cpRNFLT and HFA MD, but also cupping formation and tissue MBR. This suggests that risk factors for LC thinning, which is suspected to play a key role in glaucomatous axonal degeneration, include structural changes in optic nerve cupping and in optic nerve blood flow. The finding on blood flow is particularly significant, because blood flow has been found to be modifiable, both with glaucoma eye drops [37] and traditional Chinese medicine,[38,39] and it is the target of existing drugs and drugs currently being developed. Thus, methods to easily assess the thickness of the LC should open new avenues for the clarification of glaucoma pathogenesis, and create new fields of research into drug discovery for glaucoma treatment.
